# Vulcanite Litigation

**Published:** 1874-02

**Authors:** 


### "VULCANITE LITIGATION."

We take the following from the Feb. No. of the *Dental Cosmos.* It shows that progress is being made in the legal controversy between the Goodyear Vulcanite Company and the dental profession. We look with much interest for the decision of Judge Shepley in this case.

It will be seen that Dr. White intends soon to make some statement of his attitude as involved in this contest. This attack upon Dr. W. by the Vulcanite Company indicates a weakness and a want of reliance in the merits of their cause, of which we had not before conceived. The institution of such a suit against him proceeds, of course, upon the supposition that he has no veritable interest in the contest between that Company and the dental profession. A more false and unwarrantable supposition could hardly have been made. Dr. S. S. White was educated, under a seven years' pupilage, a dentist, is a D. D. S. and was for a long time in practice. His interest and connection with the dental profession has been and still is of the most intimate and special character. He is, and has been for many years, so far as the production of instruments, appliances and materials is concerned, of greater importance to the dental profession than any living man. He is particularly interested in, and has usually been connected with, everything that pertains to the welfare and advancement of the profession.

Now, in view of these facts, we can hardly understand how any sane man, or set of men (called a company), could conceive the idea of prosecuting for "intermeddling in a suit that in no way belongs to him, etc.," Dr. S. S. White, for the effort he is making with the dental profession against what is generally regarded as an unjust and outrageous monopoly. We have no fear for Dr. White in this matter, further than the annoyance it may cause him.

"PORTLAND, January 17th, 1874.

"On the fourteenth, fifteenth and sixteenth insts., before the Hon. George F. Shepley, United States Circuit Judge, sitting in equity at Portland, Maine, the cause of the Goodyear Dental Vulcanite Company and Josiah Bacon against Daniel H. Smith was argued. This was the case selected by the Company and Mr. Bacon from those in which were set up the defences already made familiar to the readers of the *Dental Cosmos* in the supplement to the November number. Mr. Dickinson and Mr. Lee argued the complainants' case. Mr. Baldwin presented the points of defence, and in connection therewith read and filed a written argument submitted by Judge Black.

Judge Black was unable to attend in person, by reason of engagements which detained him in Washington. Dr. Smith was also represented by E. O. Shepard, Esq., of the firm of Messrs. Jewell, Gaston and Field, of Boston, his immediate counsel of record.

The argument lasted some seventeen hours, about equally divided between the complainants and defendant.

The Judge patiently and indulgently heard the case presented at the greatest length desired by either, and for that purpose extended the time very largely beyond the limits fixed by the rules of the court. We purpose to publish at least a synopsis of the argument on both sides, but not until the decision shall have been announced. Meanwhile, we congratulate the profession upon the fact that the hearing has been had, the questions confided to a judge whose deportment and character give an impress to the argument of the cause which assures us that, whatever may be his conclusions, they will be the fair convictions of a judicial mind after careful consideration and with a clear comprehension of all the matters of fact and of law involved in the record.

When the decision will be announced we cannot conjecture; but it will, no doubt, be promptly rendered, and we can now only look for it with the solicitude which all the profession will share, and with the sanguine expectation based upon the same grounds which have always been our reliance, and which, after being argued faithfully, have been confided to a court which commands universal respect.

With the publication of the arguments we shall take occasion to make a somewhat extended statement of our own attitude in this litigation, such prominence having been given thereto by the complainants' counsel as to make it a leading feature of their side of the case; otherwise we should not feel it incumbent on us to say a word in that behalf.

Samuel S. White."

